# Backward leap technique using a novel 0.018-inch guidewire

**DOI:** 10.1055/a-2515-4007

**Published:** 2025-01-31

**Authors:** Kosuke Takahashi, Eisuke Ozawa, Yasuhiko Nakao, Masanori Fukushima, Hisamitsu Miyaaki, Kazuhiko Nakao

**Affiliations:** 1 Department of Gastroenterology and Hepatology, Graduate School of Biomedical Sciences, Nagasaki University, Nagasaki, Japan; 2 Department of Gastroenterology and Hepatology, Sasebo City General Hospital, Sasebo, Japan

Guidewire placement is the greatest challenge in endoscopic ultrasound (EUS)-guided hepaticogastrostomy (EUS-HGS) 1 2 3 4 5 . Following puncture, the guidewire often advances into the peripheral intrahepatic bile ducts, making it difficult to position the wire in the common bile duct. Here, we describe a new “backward leap technique” using a novel low-rigidity 0.018-inch guidewire ( Fig. 1 , Video 1 ) to address these challenges.

**Fig. 1:**
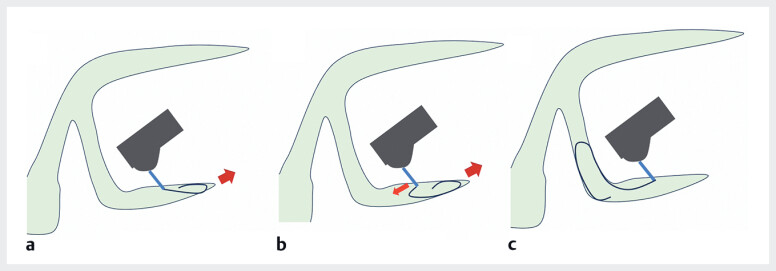
Schematic diagram of the backward leap technique using a novel 0.018-inch guidewire. **a–c** By gently advancing the low-rigidity guidewire in a looped formation toward the peripheral intrahepatic bile ducts (large arrow), the flexible portion of the guidewire near the puncture needle can be maneuvered proximally using the action–reaction principle (small arrow).

Backward leap technique using a novel 0.018-inch guidewire.Video 1

Case 1 was a 69-year-old woman who presented with obstructive jaundice caused by pancreatic head cancer and duodenal stenosis and was referred for treatment ( Fig. 2 **a** ). EUS-HGS was planned to relieve the obstruction. A 22-G needle (EZShot3Plus; Olympus, Tokyo, Japan) was used to puncture the B2 segment of the intrahepatic bile duct; however, conventional torque maneuvers failed to advance the novel 0.018-inch guidewire (Fielder 18; Olympus) into the proximal bile ducts ( Fig. 2 **b** ). Utilizing the wire’s low rigidity, a loop was formed in the peripheral bile ducts, allowing the flexible segment near the puncture needle to be maneuvered toward the hepatic hilum by applying action–reaction principles ( Fig. 2 **c** ). EUS-HGS was successfully completed without complications ( Fig. 2 **d, e** ).

**Fig. 2:**
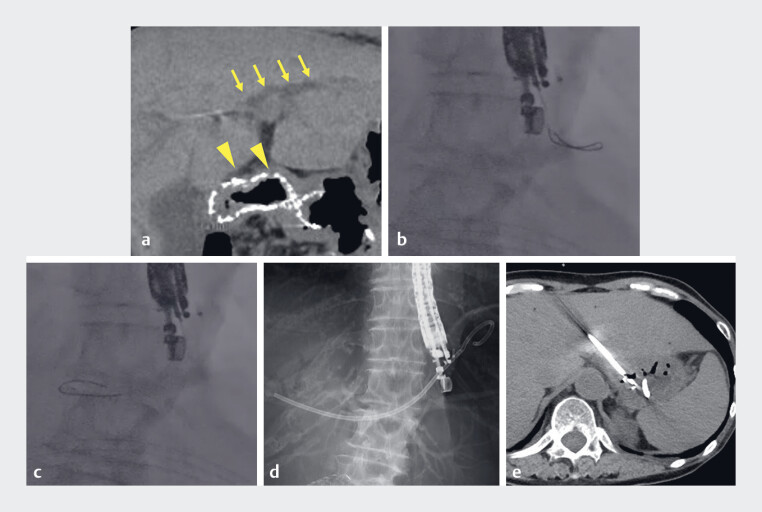
Computed tomography (CT) and cholangiography images for case 1. **a** CT image showing stenting for duodenal stenosis (arrowhead) and dilation of the left intrahepatic bile duct (arrow). **b** Cholangiography image illustrating the inability of conventional torque manipulation to advance the novel 0.018-inch guidewire to the proximal intrahepatic bile duct. **c** Cholangiography image showing the use of the backward leap technique, enabling the guidewire to maneuver toward the hilum. **d, e** Cholangiography and CT images showing successful placement of the hepaticogastrostomy stent.

Case 2 was a 43-year-old man with obstructive jaundice due to pancreatic head cancer and duodenal stenosis ( Fig. 3 **a, b** ) who also underwent EUS-HGS. During guidewire insertion, conventional torque maneuvers failed to advance the wire into the proximal bile ducts. As in case 1, by forming a loop in the peripheral ducts and utilizing the guidewire’s low rigidity, the wire was maneuvered toward the hepatic hilum ( Fig. 3 **c** ). EUS-HGS was successfully completed without any complications ( Fig. 3 **d, e** ).

**Fig. 3:**
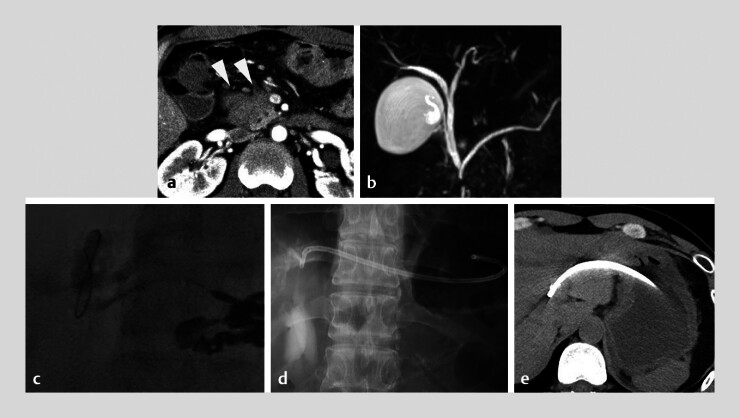
Imaging studies for case 2. **a** Computed tomography (CT) image showing a pancreatic head tumor (arrowhead). **b** Magnetic resonance cholangiopancreatography image indicating obstruction of the common bile duct and main pancreatic duct. **c** Cholangiography image demonstrating the backward leap technique, allowing the guidewire to maneuver toward the hilum. **d, e** Cholangiography and CT images showing successful placement of the hepaticogastrostomy stent.

The novel 0.018-inch guidewire, with its softer and more flexible stiff segment than conventional types, enables looping, allowing the flexible tip to “leap” backward into the hepatic hilum. This technique represents a promising new method for overcoming guidewire placement challenges in EUS-HGS.

Endoscopy_UCTN_Code_TTT_1AS_2AH

## Notes

Endoscopy E-Videos https://eref.thieme.de/e-videos
*E-Videos* is an open access online section of the journal *Endoscopy* , reporting on interesting cases and new techniques in gastroenterological endoscopy. All papers include a high-quality video and are published with a Creative Commons CC-BY license. Endoscopy E-Videos qualify for HINARI discounts and waivers and eligibility is automatically checked during the submission process. We grant 100% waivers to articles whose corresponding authors are based in Group A countries and 50% waivers to those who are based in Group B countries as classified by Research4Life (see: https://www.research4life.org/access/eligibility/ ). This section has its own submission website at https://mc.manuscriptcentral.com/e-videos .
